# Testimonial Injustice and Its Harms in Hemodialysis Patients in Japan: A Qualitative Study

**DOI:** 10.7759/cureus.106994

**Published:** 2026-04-13

**Authors:** Mariko Hokibara, Mari Tsuruwaka

**Affiliations:** 1 Bioethics/Nursing Ethics, Graduate School of Nursing Science, St. Luke's International University, Tokyo, JPN

**Keywords:** bioethics, epistemic injustice, hemodialysis, patient experience, prejudice, qualitative research, testimonial injustice

## Abstract

Objective

This study explored testimonial injustice and its associated harms arising from prejudices experienced by patients undergoing hemodialysis and examined implications for bioethics.

Methods

We conducted semi-structured interviews with 23 patients undergoing hemodialysis for one year or more. The interviews focused on the patients' experiences of prejudice, how they addressed the prejudice, and the effects of the prejudice. We analyzed the interviews by content analysis using Krippendorff's method.

Results

The analysis identified multiple forms of prejudice across both clinical and non-clinical settings. Patterns consistent with testimonial injustice were also observed, including situations in which patients were not believed or were not given opportunities to speak. In response, some participants adopted anticipatory strategies to avoid prejudice, such as withholding or modifying their testimony. These accounts correspond to testimonial smothering, in which individuals suppress their own statements in anticipation of negative evaluation. The harms identified in the data were primarily medical disadvantages, such as delayed examinations and treatment, which affected patients' access to appropriate care.

Conclusion

This study demonstrated that testimonial injustice can occur among individuals with internal disabilities whose conditions are not outwardly visible. Some patients anticipated this injustice and engaged in avoidance behaviors, such as concealing their treatment status, thereby forfeiting opportunities to be recognized as legitimate subjects of knowledge. The harms associated with these experiences included both primary and secondary forms: primary harms were identified as "invisible harms" that may threaten patients' dignity and warrant consideration under the principle of nonmaleficence, while secondary harms raised concerns from the perspective of formal justice. Addressing testimonial injustice in bioethics may help clarify how patients are harmed when their words are not taken seriously and encourage healthcare professionals to reflect on how they listen to and respond to patients.

## Introduction

Prejudice and stigma have been studied in relation to conditions such as diabetes mellitus [1-4], mental illness [5], human immunodeficiency virus (HIV) [6], and cancer [7]. Prejudice refers to judgments toward individuals or groups that are accompanied by negative emotions, whereas stigma refers to the process by which a particular attribute or condition becomes socially fixed as a "negative mark", leading individuals to be devalued and disadvantaged in their relationships and social interactions. Previous research on stigma among patients with diabetes identified perceptions such as having developed diabetes due to a lack of self-control or unhealthy behavior [3], as well as economic disadvantages such as being unable to obtain life insurance [1], decreased self-management ability and self-esteem, and negative effects on healthcare-seeking behavior [4].

In Japan, a male television presenter posted a blog about patients who developed renal failure from diabetes, stating "All self-inflicted hemodialysis patients should pay the full cost! If they cry that it's impossible, just let them die!" This comment provoked public criticism [8]. Negative perceptions, including prejudice, have been noted among healthcare providers as well, such as the view that hemodialysis patients make excessive demands and are difficult to manage [9]. Such stigma, particularly the perception that patients lack self-control and engage in unhealthy behaviors [3], may influence how clinicians assess the credibility of patients' reports regarding their symptoms or treatment adherence.

In Japan, the number of hemodialysis patients has been increasing, exceeding 340,000 as of the end of 2020 [10]. Among renal replacement therapies, hemodialysis is overwhelmingly chosen over kidney transplantation [11] or peritoneal dialysis [12], partly due to a shortage of transplant donors and the low prevalence of peritoneal dialysis. Furthermore, most medical costs are covered under Japan's universal health insurance system. Specifically, hemodialysis patients can reduce their monthly out-of-pocket limit to 10,000 yen (or 20,000 yen for high-income earners) through the High-Cost Medical Expense Benefit system [12]. Additionally, many patients meet the criteria for disability pension [13].

The first nationwide survey in Japan on prejudice and stigma among hemodialysis patients, conducted in 2021, found that about a quarter of patients felt that "dialysis is a personal responsibility" [14]. Factors such as adherence to health management behaviors, reduced sociability, and the risk of depression have been shown to influence stigma in this population [15]. Studies from China [16] and Brazil [17] have also reported the stigma experienced by hemodialysis patients, suggesting that prejudice and stigma toward this group exist regardless of country or culture.

In 2007, Fricker [18] introduced the concept of "epistemic injustice", which refers to injustice committed against someone's capacity as a knower. This includes "testimonial injustice", in which prejudice against the speaker leads to their testimony being unjustly discredited, and "hermeneutical injustice", in which the speaker's experiences are not properly interpreted. This study focuses on testimonial injustice. Fricker [18] draws on the classification of social types of prejudice, emphasizing judgments toward individuals or groups accompanied by negative affect.

Expanded concepts of epistemic injustice have also been proposed. Participatory injustice refers to exclusion from opportunities to speak or participate as a knower [19]. Testimonial smothering describes situations in which speakers, for self-protective reasons, intentionally withhold or constrain their testimony [20]. In healthcare contexts, this may occur when patients, anticipating misunderstanding or prejudice from healthcare professionals, hesitate to disclose information necessary for diagnosis or treatment.

Epistemic injustice is a concept that captures how prejudice and stigma shape the epistemic dimension of social interaction, influencing how individuals' narratives and knowledge are received and evaluated. In this study, we focus on narratives grounded in experiences of prejudice and stigma among hemodialysis patients and examine how these experiences can be understood as forms of epistemic injustice, particularly testimonial injustice.

The harm caused by testimonial injustice can be divided into primary and secondary harm [18]. Primary harm refers to the direct harm caused by being unjustly disbelieved, which damages the dignity of a person as an epistemic subject. We express our beliefs through conversations with mutual trust and shape ourselves accordingly. However, when our statements are disbelieved and excluded, the development of our identity can be hindered. Secondary harm arising from primary harm includes practical harm (e.g., the speaker being placed at a disadvantage or suffering loss in employment, social security, etc.) and epistemic harm (e.g., the speaker coming to doubt their own beliefs and losing knowledge) [21].

According to Shklar [22], philosophy has often treated injustice as a deviation from justice, yet injustice itself is deeply embedded in society. Fricker [18], drawing on this perspective, positions epistemic injustice as an issue located at the intersection of epistemology and ethics.

Testimonial injustice has four defining features: (1) it arises from prejudicial stereotypes related to social identity; (2) due to this, the listener unjustly lowers their assessment of the speaker's credibility; (3) therefore, the speaker's testimony is ignored, undervalued, or preemptively silenced, obstructing its transmission; and (4) the speaker's capacity as an epistemic subject is undermined [23]. Overcoming epistemic injustice requires first making the prejudices directed toward the speaker explicit [24].

In medical settings, patients' words can be disbelieved [25]. For example, Carel and Kidd [25] illustrate testimonial injustice in patients with both mental and physical illnesses by referring to the case of Saks [26]. Saks, who has schizophrenia, presented to the emergency department with a severe headache caused by a cerebral hemorrhage. However, her symptoms were attributed to her mental illness, and she was discharged without undergoing further examination. As a result, she did not receive appropriate treatment for the cerebral hemorrhage.

Research is emerging on the prevalence of epistemic injustice in the treatment of conditions such as psychiatric disorders [27] and chronic fatigue syndrome [28]. Empirical studies are still few, with notable examples including research on female surgeons [29] and individuals experiencing auditory hallucinations [30]. No previous studies examined hemodialysis patients.

Previous work on bioethics and epistemic injustice has argued from the perspective of nonmaleficence, among the four principles of biomedical ethics, that it can harm patients' dignity and the formation of their identity [31]. Given the scarcity of studies that elucidate the prejudice experienced by hemodialysis patients from their perspective and the absence of research focusing specifically on testimonial injustice and its harm, this study aimed to examine the prejudice and testimonial injustice experienced by hemodialysis patients.

Prejudice and stigma toward hemodialysis patients are formed not only within clinical settings but also across everyday life contexts. Accordingly, this study elicited experiences of prejudice in both clinical and non-clinical settings and examined how these experiences shaped credibility-related interactions with healthcare professionals, for example, when patients are perceived as having poor self-management, which may influence how clinicians assess or respond to patients' accounts of their symptoms or adherence, including experiences that may reflect testimonial injustice and its harms.

Existing research on epistemic injustice in healthcare has largely relied on case-based analyses, with limited empirical investigation [32]. To address this gap, the present study examines testimonial injustice grounded in patients' experiences of prejudice and offers insights for fostering better patient-healthcare professional relationships. Doing so can contribute to bioethics research by offering insights into the nature of patient-provider communication and ethical considerations.

Therefore, this study aimed to explore testimonial injustice and its associated harms in relation to prejudice experienced by hemodialysis patients and to provide insights for addressing these issues within bioethics.

## Materials and methods

Study design

This study employed a qualitative descriptive design and was conducted and reported in accordance with the Consolidated Criteria for Reporting Qualitative Research (COREQ) [33]. Data were collected through semi-structured interviews conducted either face-to-face or online, each lasting approximately 60-90 minutes. Data were collected from June to August 2024. The basic demographic and clinical attributes collected included age, sex, family background, occupation, medical history, and the duration and course of hemodialysis since initiation.

There are very few qualitative studies on epistemic injustice in healthcare. Therefore, we developed an original interview guide to explore prejudice-related experiences across everyday life contexts and to examine how some narratives may reflect testimonial injustice and its harms. In developing the interview guide, we drew on methodological approaches used in prior qualitative research examining bias and credibility in healthcare, including a study on gender bias among female surgeons [29]. While the guide was adapted to the context of hemodialysis patients and the aims of the present study, this prior work informed the framing of open-ended questions designed to elicit experiences of prejudice, dismissal, or not being taken seriously across various contexts. An English version of the interview guide is provided in the Appendices.

The interview guide for this study was developed based on the premise that prejudice toward hemodialysis patients is formed not only in clinical settings but also across everyday life contexts. Experiences of testimonial injustice are not necessarily recognized consciously by hemodialysis patients, and formulating interview questions that presuppose this concept risks intentionally steering participants' narratives into a predefined theoretical framework.

Therefore, this study did not employ questions designed to directly identify testimonial injustice. Instead, interview questions were deliberately framed broadly to allow participants to freely describe their experiences of prejudice in everyday life. Through analysis of these narratives, we examined how participants' accounts reflected experiences of credibility deficit, dismissal, and self-protective silence and how these experiences may be interpreted within the framework of testimonial injustice. In particular, participants were encouraged to recall experiences in both clinical and non-clinical settings. Non-clinical settings included family contexts, workplace contexts, friendships and broader social relationships (beyond romantic relationships), and wider societal situations such as community and public interactions. Participants were asked about the prejudices they had experienced in various contexts using the following prompt: "Please recall and describe any negative views (prejudices) you have experienced from others in any aspect of your life (healthcare, work, family, relationships with friends and acquaintances, or broader social interactions such as community and public settings). Please provide specific examples of situations in which you felt prejudiced due to undergoing hemodialysis. At that time, what did you feel, how did you respond, and what impacts did you experience?"

Participants and recruitment

Participants were hemodialysis patients who met the following inclusion criteria and provided informed consent: they were adults aged 18 years or older, had undergone hemodialysis for at least one year, were able to communicate verbally in Japanese, and had no restriction regarding the type of primary disease. The exclusion criteria were patients undergoing home hemodialysis, those with reduced cognitive function, and those who were currently hospitalized.

Recruitment was conducted via three routes: First, department heads (physicians) and nursing managers at dialysis clinics or hospitals were asked to identify patients who met the inclusion criteria. Second, leaders of associations for hemodialysis patients were asked to disseminate information regarding the study. Third, through the researcher's personal network of healthcare professionals, introductions to hemodialysis patients were obtained, after which additional participants were recruited via snowball sampling.

Data analysis

We used Krippendorff's content analysis [34] to conduct a systematic analysis. An inductive approach was used to derive categories and subcategories from participants' narratives.

First, verbatim transcripts were prepared from the audio recordings and notes, and each case was read repeatedly to gain an overall understanding of the narrative, including its structure and context. Subsequently, segments related to participants' experiences of prejudice, their coping responses, instances of testimonial injustice where their accounts were not acknowledged by others, and the consequences of these experiences were identified. Within each context, experiences were further examined in relation to clinical and non-clinical settings. Codes were generated from these segments while preserving the context of each narrative. Finally, codes were compared to identify similarities and differences and were grouped into subcategories and categories through abstraction without losing their original meaning.

Reflexivity

The first author conducted all recruitment and interviews and led the primary analysis. Given the interpretive nature of qualitative research, reflexive consideration was maintained throughout the study to examine how the researcher's assumptions and perspectives might influence data collection and interpretation. Field notes and analytic memos were used to support reflexive engagement during the analytic process.

To minimize the risk that the analysis reflected the perspective of a single researcher, the co-author (supervising professor), who is an expert in bioethics and experienced in qualitative research, independently coded all transcripts. Thus, coding was conducted independently by two researchers. The interpretations and coding framework developed by the first author were compared with those of the co-author. When discrepancies could not be resolved through initial discussion, both researchers revisited the data and conducted further independent analysis before refining the coding framework. This iterative process strengthened reflexivity and supported the credibility of the findings. Care was taken to ensure that the interpretations remained grounded in participants' accounts and reflected their lived experiences.

Ensuring rigor

To enhance credibility and dependability, the analytic process was documented and discussed repeatedly among the authors throughout the study. Categories and subcategories were continuously refined through comparison across cases, and interpretations were checked against the original transcripts to ensure that findings were grounded in participants' accounts. The authors examined alternative interpretations during analytic discussions to reduce premature closure and strengthen analytic rigor.

Member checking was not conducted in this study due to concerns about placing additional burden on hemodialysis patients, who may experience physical strain during treatment and may also have work-related commitments. Instead, the credibility of the analysis was supported through independent coding by two researchers and iterative discussions to refine the interpretations.

Ethical considerations

Participants were informed in writing about the study purpose, methods, protection of personal information, the voluntary nature of participation, and data management. Written informed consent was obtained from all participants. This study was approved by the Research Ethics Committee of St. Luke's International University (approval number: 24-A006). All procedures were conducted in accordance with the principles of the Declaration of Helsinki.

## Results

Participants were 23 individuals undergoing hemodialysis (19 men and four women), reflecting the male-to-female ratio among hemodialysis patients in Japan [35]. Participants ranged in age from 36 to 73 years, and more than half were in their 30s to 50s. Primary diseases included chronic glomerulonephritis, diabetic nephropathy, and nephrosclerosis. Approximately 70% of participants were employed. Detailed demographic and clinical characteristics are shown in Table 1.

**Table 1:** Demographic and clinical characteristics of participants (N=23)

Demographic characteristics		Number	%
Sex	Male	19	82.6
	Female	4	17.4
Age group (years)	30s	3	13
	40s	2	8.7
	50s	8	34.8
	60s	5	21.7
	70s	5	21.7
Primary disease	Chronic glomerulonephritis	7	30.4
	Diabetic nephropathy	6	26.1
	Nephrosclerosis	6	26.1
	Pediatric chronic kidney disease	2	8.7
	Lupus nephritis	1	4.3
	Polycystic kidney disease	1	4.3
Years on hemodialysis	1-4 years	7	30.4
	5-9 years	9	39.1
	10-14 years	2	8.7
	15-19 years	1	4.3
	20-24 years	4	17.4
Employment status	Full-time permanent employee	7	30.4
	Self-employed	6	26.1
	Unemployed (including retired)	6	26.1
	Part-time or freelance	4	17.4

Prejudice experienced by hemodialysis patients

Eight categories and 28 subcategories were identified for the prejudice experienced by hemodialysis patients, and five categories and 10 subcategories were identified for coping strategies against prejudice (Table 2). In this study, "non-clinical settings" refers to everyday life contexts outside healthcare, including interactions with family members, colleagues, friends, and acquaintances, as well as broader societal situations such as community and public settings.

**Table 2:** Prejudice experienced and coping strategies of hemodialysis patients

Setting	Category	Subcategory
Non-clinical settings	Hemodialysis is seen as self-inflicted	Due to overeating, drinking, and unhealthy lifestyle habits
		Hemodialysis = diabetes
	Seen as "incapable"	Cannot enjoy social gatherings
		Cannot live long
		Can hardly eat or drink
		Cannot work overtime or full-time
		Cannot exercise
		Cannot travel due to time restrictions
	Looked at with pity	Surgical scar from shunt operation seen as a self-harm scar
		Shunt bulge looked at with curiosity
		Seen as a difficult person defined by "illness" or "being a patient"
	Being regarded as incapable due to being seen as "seriously ill"	Life is over; cannot do anything
		Cannot be promoted
		Cannot work properly
		Considered a difficult partner to marry
	Not being seen as "ill" due to internal disability	Viewed more negatively compared to those with visible disabilities
		Considered not needing consideration for a health condition
		Leaving work early for dialysis is not considered justified
		Not recognized as having a disability even when presenting a disability certificate
		Not regarded as needing consideration even when wearing a help mark
Clinical settings	Being regarded as a low-urgency patient	Less urgent than other acute care areas
		Considered a patient who can be handled routinely
		Pain complaints are not taken seriously
	Hemodialysis patients regarded as selfish	Considered a selfish patient
		Considered a nagging patient
Non-clinical settings	Being regarded as favored by public assistance	Using too much medical expense
		Using too much tax money
		Companies and hospitals can take advantage of disability employment/medical cost exemption systems
Non-clinical settings	Hiding the shunt	Not disclosing undergoing hemodialysis
		Disclosing undergoing hemodialysis
	Remaining silent in advance	Not telling to avoid impact on work
		Not telling to avoid being treated as sick
	Pretending to be healthy while undergoing medical treatment	Conveying in a downplayed manner
		Conveying something untrue
	Disclosing selectively	Telling when necessary for daily life
		Telling based on the other person's personality or trustworthiness
	Actively disclosing	Not hiding the disease name
		Deliberately showing the shunt

Non-clinical Settings

Being regarded as personally responsible for undergoing hemodialysis: In non-clinical settings, including workplace environments and interactions with friends and acquaintances, participants described being blamed for their need for hemodialysis, regardless of their primary disease. Dialysis was frequently equated with unhealthy lifestyle behaviors and framed because of personal failure rather than a medical condition. As one participant explained: "My supervisor and colleagues said things like, 'It's your own fault-you brought it on yourself because you couldn't manage your health properly'" (Participant 19). 

Such remarks were described by multiple participants in workplace settings and in interactions with friends and acquaintances. Participants reported feeling that hemodialysis was viewed as a matter of personal fault rather than a medical condition and that this perception affected how they were treated in subsequent interactions.

Being broadly interpreted as "incapable": Participants described being viewed by others as broadly incapable in everyday life, particularly in workplace contexts and interactions with friends and acquaintances. They reported that others assumed they were unable to enjoy social gatherings, faced severe dietary restrictions, could not work full-time, or were unable to travel because of dialysis schedules. One participant recalled being told by an acquaintance: "You're not allowed to eat things like fruit or vegetables, right?" (Participant 7).

Similar remarks were described by other participants. Participants reported feeling that others made assumptions about the limits of their daily activities and future prospects without asking about their actual capabilities or preferences.

Being looked upon with pity: Several participants described being viewed with pity in everyday social interactions because of visible signs associated with hemodialysis treatment. They reported being stared at in public spaces, such as on the street or on public transportation. Surgical scars from arteriovenous shunt creation were sometimes mistaken for self-harm scars, and the bulging of the shunt site attracted attention.

One participant described how a relative reacted upon noticing the shunt: "A relative once said, 'What happened to your arm?' And when I told them I was on dialysis, their reaction changed to something like, 'Oh… you're on dialysis…'" (Participant 15).

The participant described that the atmosphere shifted after this exchange and that the way they were looked at felt different from before.

Some participants expressed that, after others became aware of their treatment, they were seen primarily in relation to their illness. As one participant stated: "Everything becomes based on the illness and turns into pity" (Participant 13).

Being regarded as incapable due to being seen as "seriously ill": Participants described being perceived as seriously ill to the extent that their abilities in work and social life were questioned. Such perceptions emerged in conversations with family members, colleagues, business contacts, friends and acquaintances, romantic partners, and even strangers in public settings. Some participants reported that others spoke as if their productive life had effectively ended after starting dialysis. One participant recalled overhearing a conversation between a couple who were touring the hospital and happened to be present in the dialysis unit: "Dialysis… once you end up in that, your life is basically over. You have to go to the hospital three times a week for hours, right? If you become like that, your life is over" (Participant 16).

Participants described that comments such as these made them feel that their future was being spoken about in fixed and limited terms. Others reported being treated as unsuitable for promotion at work or as undesirable partners for marriage after their dialysis status became known.

Not being seen as ill due to having an internal disability: Participants described not being recognized as ill because hemodialysis constitutes an internal (invisible) disability. In workplace contexts and on trains, they reported being treated as healthy and not in need of accommodation, as their condition was not outwardly visible.

Several noted that unless they disclosed their illness, such as by showing their arteriovenous shunt, they were not treated as patients undergoing hemodialysis because they "looked healthy". In Japan, participants referred to a "Help Mark", a widely recognized symbol for invisible disabilities that can be carried as a tag or badge to indicate that a person may require consideration in public settings. However, they reported that this did not consistently change others' responses. As one participant stated: "The help mark is basically meaningless. People almost never give up their seat on the train" (Participant 1).

Being regarded as favored by public assistance: Participants described perceiving a broader social climate in which hemodialysis patients were viewed as unfairly benefiting from public assistance because of the high costs associated with long-term treatment. Rather than referring to a single individual, participants reported sensing such attitudes through everyday interactions, including workplace contexts and their experiences within healthcare institutions. One participant explained, "People say things like, 'Dialysis patients use so much tax money, so you should pay for it yourself'. I feel like society is starting to move in that direction" (Participant 9).

Participants also described situations in which the medical cost exemption system led others to assume that they could freely access healthcare services without financial constraints. For example, one participant recalled being repeatedly encouraged to undergo additional dental procedures: "When I went to the dentist, they kept saying, 'Why don't you do this as well? And that too? It's free anyway'" (Participant 19).

Such experiences were described as leaving participants with the impression that they were perceived as "taking advantage" of public support and that their healthcare use was viewed as excessive or unnecessary.

Clinical Settings

Being regarded as a patient with low urgency: Within clinical settings, participants perceived that hemodialysis care was treated as less urgent than other medical areas, particularly because many patients appeared clinically stable and attended treatment on a regular basis. As a result, participants felt that their needs and concerns were less likely to receive close attention from physicians.

One participant described that dialysis units were often operated primarily by dialysis technicians (clinical engineers in Japan) and nurses, with limited involvement of full-time physicians. The participant stated: "Most hemodialysis clinics can basically be run by dialysis technicians and nurses. In that hospital, the full-time nephrologists were not involved at all. There was no full-time physician assigned to the dialysis unit-it was left to part-time doctors" (Participant 21).

The participant further explained that this organizational structure reflected an implicit perception that hemodialysis was not considered an urgent field within the hospital system: "I think the hospital and doctors see dialysis as less urgent than other areas. That's why the unit is mostly managed by dialysis technicians and nurses" (Participant 21).

Such experiences contributed to participants' impressions that they were treated as routine cases rather than as patients requiring individualized medical attention.

Some participants also reported that even when they complained of pain, such as during shunt puncture, their concerns were not taken seriously, reinforcing the sense that their suffering was minimized.

Being labeled as "selfish": Some participants described experiences in which healthcare professionals portrayed hemodialysis patients in a negative light, particularly by characterizing them as "selfish". One participant recalled that during hospitalization, nurses repeatedly made remarks suggesting that dialysis patients were selfish: "When I was hospitalized, the nurses all said, 'Dialysis patients are all selfish'. I can't forget it. They said, 'Dialysis patients are really selfish'" (Participant 8).

Such remarks led participants to feel stigmatized not only as patients requiring long-term treatment but also as individuals who were judged in terms of their character within the clinical setting. Another participant described feeling negatively labeled when actively seeking information about treatment options. When asking nurses about home dialysis, the participant felt they were viewed as troublesome: "Because I often asked the nurses about home dialysis, I felt they probably saw me as someone who is picky or overly sensitive" (Participant 23).

Coping strategies for prejudice experienced by hemodialysis patients

Non-clinical Settings

Hiding the shunt: In response to anticipated prejudice from others, participants described efforts to conceal visible signs of hemodialysis treatment, particularly their shunts. Several participants reported wearing long sleeves even in summer to avoid being visually identified as dialysis patients. One participant explained, "I always wear long sleeves even in summer because I'm conscious of how people look at me. I would have to explain why I'm on dialysis, and since it's due to my own poor lifestyle, I'd rather not talk about it" (Participant 4).

Even among participants who disclosed that they were undergoing hemodialysis, some continued to hide their shunts, indicating persistent concern about being judged based on visible bodily markers.

Remaining silent in advance: Another strategy involved withholding information about their condition in advance to avoid being treated as ill. Participants described deliberately not disclosing their hemodialysis status in professional contexts to prevent negative assumptions about their health or work performance. As one participant stated, "I try not to tell clients about my illness. If I do, they'll think that because I'm on dialysis my health is bad and it will affect my work" (Participant 15).

These accounts indicate that participants anticipated a loss of trust or credibility if their condition became known.

Pretending to be healthy while undergoing medical treatment: Some participants described downplaying the burden of hemodialysis in order to avoid attracting attention or concern. This included providing simplified explanations that framed dialysis as less serious than it actually was. One participant recounted an interaction with a supervisor, saying, "When my boss says dialysis must be tough, I shouldn't lie, but I tell them it's just over two hours and like an IV drip" (Participant 23).

Such statements reflect attempts to manage others' perceptions by presenting dialysis as a routine and manageable treatment.

Disclosing selectively: Selective disclosure was commonly described. Participants reported carefully choosing when and to whom they disclosed their hemodialysis status, depending on practical necessity and the nature of their relationships. One participant explained: "Since I run a company, the people at work know because I sometimes have to leave early or adjust my schedule for dialysis. My husband and his family know, and three very close friends know. But I haven't told anyone else" (Participant 10).

Participants described limiting disclosure to those directly involved in their daily routines or intimate relationships while withholding information from others.

Actively disclosing: In contrast, a smaller number of participants described active disclosure strategies, in which they intentionally did not conceal their diagnosis and, in some cases, openly displayed their shunts. One participant stated, "As a company president, when new employees join, I explain dialysis to them in detail. If you explain, everyone understands" (Participant 3).

This approach was described to prevent misunderstanding and reduce prejudice through proactive communication.

Testimonial injustice and associated harms 

Analysis of participants' narratives revealed experiences in which their credibility was undermined and their accounts were dismissed or disregarded in both clinical and non-clinical contexts. These experiences can be interpreted as reflecting testimonial injustice, as participants described credibility deficits rooted in prejudice and reported harms they associated with these experiences. One category, "diminished credibility", comprising two subcategories and six codes, was identified. Table 3 presents representative contexts illustrating testimonial injustice and its associated harms.

**Table 3:** Testimonial injustice and associated harms hANP: human atrial natriuretic peptide

Category	Subcategory	Illustrative cases	Setting	Harms
Diminished credibility	Not being believed	Explanation that the participant could balance hemodialysis and work was not believed by clients	Non-clinical	Lost approximately half of their clients after disclosing the illness
		Repeatedly reported appetite loss and weight loss, but concerns were not addressed by the physician	Clinical	Changed hospitals due to unresolved symptoms
		Repeatedly consulted about headaches, but no measures were proposed by the physician	Clinical	Changed clinics due to persistent headaches
		Reported pain during puncture, but healthcare professionals did not take the complaint seriously	Clinical	Became reluctant to speak openly to healthcare providers in order to continue attending the same dialysis facility
		Requested an hANP test to reassess dry weight, but the physician did not order the test	Clinical	Experienced delayed symptom improvement due to delayed adjustment of dry weight
	Not being given an opportunity to speak	Denied an opportunity to explain after the fiancé's parents opposed the marriage due to the participant's hemodialysis status	Non-clinical	Became reluctant to pursue future romantic relationships or marriage

Diminished credibility

Within this category, participants reported situations in which they perceived that prejudice contributed to a credibility deficit, which they perceived as leading to their accounts being dismissed or disregarded. Many participants described experiences consistent with testimonial injustice and its associated harms, which appeared to occur more frequently in clinical settings than in non-clinical contexts.

Not being believed

Non-clinical Settings

One participant who returned to work after initiating hemodialysis described difficulties in interactions with clients. Despite explaining that dialysis treatment could be balanced with work, the participant felt their explanation was repeatedly dismissed: "Even when I explained to clients that I could balance dialysis treatment and work, I sometimes got responses like, 'I'll ask you once you're cured', or, 'Then I won't bother asking you'. Clients didn't understand even when I explained; they just didn't listen. They weren't interested in listening, so they'd say things like, 'Come back when you're cured'. When I told them my illness wouldn't be cured, they said, 'Then never mind, I'll ask someone else'. I felt they didn't understand or listen to me, and my clients dropped to about half" (Participant 1).

The participant reported that he lost about half of their clients after disclosing their hemodialysis status.

Clinical Settings

In clinical settings, participants described similar experiences when reporting physical symptoms. One participant reported severe appetite loss after starting dialysis, which was accompanied by significant weight loss: "I repeatedly told the dialysis unit physician that I wanted them to do something about it. But the doctor just said, 'If you have no appetite, you don't have to eat'. No matter how many times I said it, nothing happened, and I lost trust in the doctor, so I changed hospitals" (Participant 21).

After changing hospitals, the participant was found to have zinc deficiency, and supplementation improved their symptoms.

Another participant described repeatedly raising concerns about headaches without receiving any response from the physician: "Though I discussed my headaches each time with the clinic doctor, that was the end of it. They didn't think about ways to improve things. So I changed clinics" (Participant 5).

After switching clinics, the participant reported that increasing the dialysis duration from four to five hours led to symptom improvement.

In another clinical interaction, one participant reported pain caused by the tight application of a tourniquet during puncture. When expressing this pain, the participant felt their complaint was minimized: "The medical staff told me, 'Pain is unavoidable'. I felt they thought, 'You're being selfish'. After that, because I had to keep going to the same facility, I started holding back and found it harder to say what I wanted to the staff" (Participant 23).

Following this experience, the participant became reluctant to speak openly to healthcare providers in order to maintain the ongoing treatment relationship.

Finally, a participant who requested additional testing due to feeling unwell described not being taken seriously: "I told the doctor I felt bad and asked them to check things like my dry weight settings and hANP, but they didn't. Later, when they finally checked, my hANP was very low, and my dry weight had changed. It was a simple matter. I think that doctor didn't take my words seriously and saw me as a 'complaining patient'. They probably found me 'annoying'" (Participant 21).

The participant reported that delays in testing and dry weight adjustment contributed to prolonged symptoms.

Not being given an opportunity to speak

Non-clinical Settings

One participant described an experience involving marriage intentions. Although the fiancé's parents initially approved of the relationship, they opposed the marriage after learning about the participant's disability: "When we first met, I had received their approval for marriage. But once they became aware of my disability, they opposed the marriage, and we broke up. I couldn't explain to my partner's parents because they wouldn't meet me. It broke my heart. Difficulties in relationships went up after that" (Participant 18).

Following this experience, the participant described becoming hesitant about pursuing future romantic relationships or marriage.

## Discussion

Testimonial injustice experienced by patients with invisible disabilities

The results showed that hemodialysis patients experienced various forms of prejudice not only in medical settings but also in their daily lives. The perception that hemodialysis is the patient's responsibility aligns with previous findings among patients with type 2 diabetes [3]. Similarly, the view held by healthcare providers that hemodialysis patients are "complaining" or "selfish" [9] was supported by participants' narratives in this study.

This study also identified several aspects of prejudice that have not been sufficiently highlighted in previous research on hemodialysis patients, including overgeneralization of lifestyle restrictions, pity directed toward arteriovenous shunts, being treated as a "non-patient", being regarded as low-urgency patients, and being perceived as unduly benefiting from public assistance. Moreover, these experiences were consistent with testimonial injustice, as participants described credibility deficits rooted in prejudice and the dismissal of their accounts. To our knowledge, no previous empirical study has explicitly examined testimonial injustice and its harms among hemodialysis patients; thus, the present study contributes an empirical example of this concept in the context of long-term dialysis care.

Fricker [18] originally discussed epistemic injustice mainly in relation to social identities with visible markers, such as race and gender, and later noted that epistemic injustice may also occur in patients [36]. However, hemodialysis patients often have "invisible disabilities", and their patient status may not be readily apparent, particularly when bodily signs such as shunts are concealed. The findings of this study suggest that even patients with invisible disabilities may experience testimonial injustice, whereby prejudice held by listeners contributes to diminished credibility and disbelief in patients' statements.

Anticipating and avoiding testimonial injustice

Previous research has shown that some patients with type 2 diabetes choose not to disclose their illness as a strategy for coping with stigma [3]. Similarly, participants in this study described managing information about their hemodialysis status in order to avoid anticipated prejudice. This included intentionally refraining from disclosure, concealing their shunts, and downplaying or simplifying explanations regarding dialysis schedules or treatment details.

These narratives suggest that some participants anticipated being misunderstood or negatively evaluated if their hemodialysis status became known. Although such experiences may not always involve explicit acts of disbelief, they indicate that concerns about credibility and negative interpretation shaped participants' communication in advance.

In this respect, the concept of testimonial smothering, as articulated by Dotson [20], provides a useful theoretical lens. Testimonial smothering refers to situations in which speakers anticipate that their testimony will not be understood or will be treated inappropriately and therefore intentionally withhold, constrain, or modify what they might otherwise say. The narratives in this study align with this concept, as participants described suppressing or shaping their disclosures in anticipation of negative reactions.

While these strategies may function as self-protective responses, they may also constrain patients' opportunities to be recognized as credible knowers. The findings therefore suggest that epistemic injustice in healthcare may operate not only through overt acts of disbelief but also through anticipatory silencing that limits patients' epistemic agency.

Harms resulting from testimonial injustice

Participants described experiences in which their ability to balance work and hemodialysis was not believed, as well as situations in which marriage was opposed because of their hemodialysis status and they were denied an opportunity to explain. These experiences may constitute primary harm, in that patients' dignity and status as credible speakers were undermined.

Previous research on prejudice and stigma has mainly reported interpersonal and occupational impacts and, in medical contexts, reduced self-management such as medication adherence [14]. In contrast, this study identified additional harms related to healthcare access and outcomes. Participants reported that inadequate responses to physical symptoms led to transfers to other medical facilities and delayed symptom improvement, suggesting that dismissal of patients' reports may result in secondary medical disadvantages. Occupational and interpersonal disadvantages described in this study can also be understood as forms of secondary harm associated with testimonial injustice, as discussed by Fricker [18].

The need to address testimonial injustice as an issue in bioethics

Della Croce [31] has argued that testimonial injustice causes a form of moral harm when patients' capacities as knowers are undervalued, even if this harm is not immediately visible. This argument is particularly relevant to the bioethical principle of nonmaleficence, because it highlights that harm can occur not only through physical injury but also through the denial of patients' epistemic standing. In the present study, participants described experiences in which their testimony was not believed or they were denied an opportunity to explain their circumstances. These accounts suggest the presence of primary harm, insofar as patients were not recognized as credible knowers and experienced a threat to their dignity. This finding underscores the importance of considering testimonial injustice as a form of harm that should be addressed within the framework of nonmaleficence.

The findings also indicate that testimonial injustice is closely related to justice in bioethics. Justice is often discussed in terms of distributive justice; however, the present findings are better understood from the perspective of formal justice, namely, the principle that "equals should be treated equally". From this viewpoint, patients who report physical symptoms such as headaches or appetite loss should be taken seriously and receive appropriate clinical responses regardless of whether they are receiving hemodialysis. However, participants described that their symptoms were dismissed because healthcare providers assumed that their general condition was stable or that hemodialysis patients were less urgent than those in other clinical areas. Such credibility deficits may result in delayed examinations, insufficient treatment, persistent symptoms, and, in some cases, transfers to other facilities. These consequences can be understood as secondary harms of testimonial injustice, because the initial dismissal of patients' testimony may lead to concrete medical disadvantages.

By addressing testimonial injustice within bioethics, it becomes possible to make visible forms of harm that are otherwise overlooked in clinical ethics discussions. Furthermore, examining testimonial injustice may help clarify how prejudicial assumptions shape patient-provider communication and influence the quality of care. The experiences of hemodialysis patients described in this study therefore provide an important perspective for understanding ethical challenges in patient-provider relationships, particularly in contexts of chronic illness where patients repeatedly interact with healthcare professionals over long periods.

Limitations and future directions

This study examined testimonial injustice and its associated harms as described by hemodialysis patients; however, the analysis was limited to patients' perspectives. The association between testimonial injustice and specific medical outcomes, such as delayed treatment, is based on participants' perceptions rather than objective clinical measures and should therefore be interpreted with caution. Future research should incorporate both patients' and listeners' perspectives, including those of healthcare professionals, to further elucidate testimonial injustice in this context.

Participant selection by department heads and nursing managers may have introduced selection bias.

As this study was conducted in Japan, the transferability of the findings is limited. By accumulating further cases, it may be possible to reach conceptual saturation and enhance the applicability of the findings across diverse contexts.

## Conclusions

This study suggested that various forms of prejudice and testimonial injustice may occur and may result in harm, through narratives describing prejudice experienced by hemodialysis patients. These findings suggest that testimonial injustice may arise not only in relation to visible characteristics but also among patients with invisible disabilities. By analyzing coping strategies in relation to testimonial injustice, this study suggests that even when testimonial injustice appears absent, patients may manage their identities to avoid it, indicating that assessing only its presence or absence is insufficient. Both primary and secondary harms associated with testimonial injustice were observed. In particular, secondary harms include medical disadvantages. From a bioethical perspective, this raises concerns related to the principle of justice, as illnesses may be overlooked, and also highlights implications for the principle of nonmaleficence. Understanding patients' experiences is essential for healthcare professionals in building patient-provider relationships, and such understanding may contribute to addressing testimonial injustice. This also highlights the importance of epistemic humility in clinical practice.

## Appendices

Interview guide

1. Participant Characteristics

(a) Could you please tell me about your age and gender?

(b) Could you describe your family background?

(c) What is your current occupation?

(d) Could you tell me about your medical history?

(e) How long have you been undergoing hemodialysis treatment?

(f) Could you describe the circumstances that led to the initiation of hemodialysis?

2. Experiences of Prejudice in Everyday Life

(a) Please recall and describe any negative views or prejudices you have experienced from others in any aspect of your life (e.g., healthcare, work, family, social relationships, or society).

3. Prejudices Related to Hemodialysis

(a) Please describe specific situations in which you felt prejudiced because of undergoing hemodialysis.

4. Reactions and Impacts

(a) At that time, what did you feel?

(b) How did you respond?

(c) What specific impacts, if any, did you experience?
